# *Sensors* Best Paper Award 2015

**DOI:** 10.3390/s150102228

**Published:** 2015-01-14

**Authors:** Vittorio M.N. Passaro, W. Rudolf Seitz, Assefa M. Melesse, Alexander Star, Leonhard Reindl

**Affiliations:** 1 Department of Electrical and Information Engineering, Politecnico di Bari, Via E. Orabona n. 4, 70125 Bari, Italy; E-Mail: vittorio.passaro@poliba.it; Tel.: +39-80-5963-850; Fax: +39-80-5963-410; 2 Analytical Chemistry, Department of Chemistry, University of New Hampshire, Durham, NH 03824-3598, USA; E-Mail: wrs@cisunix.unh.edu; Tel.: +1-603-862-2408; Fax: +1-603-862-4278; 3 Department of Earth and Environment, AHC-5-390, Florida International University, 11200 SW 8th Street, Miami, FL 33199, USA; E-Mail: melessea@fiu.edu; Tel.: +1-305-348-6518; Fax: +1-305-348-3877; 4 Department of Chemistry, University of Pittsburgh, 219 Parkman Avenue, Pittsburgh, PA 15260, USA; E-Mail: astar@pitt.edu; Tel.: +1-412-624-6493; Fax: +1-412-624-4027; 5 Laboratory for Electrical Instrumentation, Department of Microsystems Engineering - IMTEK, Faculty of Engineering, Albert-Ludwigs-University of Freiburg, Georges-Koehler-Allee 106, D-79110 Freiburg, Germany; Tel.: +49-761-203-7220; Fax: +49-761-203-7222

Since 2011, an annual award system was instituted to recognize outstanding *Sensors* papers that are related to sensing technologies and applications and meet the aims, scope and high standards of this journal [1–4]. This year, the winners were chosen by the Section Editor-in-Chiefs of *Sensors* from among all the papers published in 2011 to track citations. Reviews and full research articles were considered separately. We gladly announce that the following eight papers were awarded the *Sensors* Best Paper Award in 2015.

**Article Award:**

1^st^ Prize

**Ellen L. Holthoff, Dimitra N. Stratis-Cullum and Mikella E. Hankus**

A Nanosensor for TNT Detection Based on Molecularly Imprinted Polymers and Surface Enhanced Raman Scattering

*Sensors* **2011**, *11*(3), 2700-2714; doi:10.3390/s110302700

Available online: http://www.mdpi.com/1424-8220/11/3/2700

2^nd^ Prize

**Ye Tian, Wenhui Wang, Nan Wu, Xiaotian Zou and Xingwei Wang**

Tapered Optical Fiber Sensor for Label-Free Detection of Biomolecules

*Sensors* **2011**, *11*(4), 3780-3790; doi:10.3390/s110403780

Available online: http://www.mdpi.com/1424-8220/11/4/3780

3^rd^ Prize

Michele D. Kattke *, Elizabeth J. Gao, Kim E. Sapsford, Larry D. Stephenson and Ashok Kumar

FRET-Based Quantum Dot Immunoassay for Rapid and Sensitive Detection of *Aspergillus amstelodami*

*Sensors* **2011**, *11*(6), 6396-6410; doi:10.3390/s110606396

Available online: http://www.mdpi.com/1424-8220/11/6/6396

4^th^ Prize

**Jordi Llorens, Emilio Gil, Jordi Llop and Alexandre Escolà**

Ultrasonic and LIDAR Sensors for Electronic Canopy Characterization in Vineyards: Advances to Improve Pesticide Application Methods

*Sensors* **2011**, *11*(2), 2177-2194; doi:10.3390/s110202177

Available online: http://www.mdpi.com/1424-8220/11/2/2177

5^th^ Prize

**Constantin Apetrei, Irina Mirela Apetrei, Jose Antonio De Saja and Maria Luz Rodriguez-Mendez**

Carbon Paste Electrodes Made from Different Carbonaceous Materials: Application in the Study of Antioxidants

*Sensors* **2011**, *11*(2), 1328-1344; doi:10.3390/s110201328

Available online: http://www.mdpi.com/1424-8220/11/2/1328

**Review Award:**

1^st^ Prize

**Sookyoung Roh, Taerin Chung and Byoungho Lee**

Overview of the Characteristics of Micro- and Nano-Structured Surface Plasmon Resonance Sensors

*Sensors* **2011**, *11*(2), 1565-1588; doi:10.3390/s110201565

Available online: http://www.mdpi.com/1424-8220/11/2/1565

2^nd^ Prize

**Alphus D. Wilson and Manuela Baietto**

Advances in Electronic-Nose Technologies Developed for Biomedical Applications

*Sensors* **2011**, *11*(1), 1105-1176; doi:10.3390/s110101105

Available online: http://www.mdpi.com/1424-8220/11/1/1105

3^rd^ Prize

**Elizabeth A. Baldwin, Jinhe Bai, Anne Plotto and Sharon Dea**

Electronic Noses and Tongues: Applications for the Food and Pharmaceutical Industries

*Sensors* **2011**, *11*(5), 4744-4766; doi:10.3390/s110504744

Available online: http://www.mdpi.com/1424-8220/11/5/4744

These eight exceptional papers are valuable contributions to *Sensors* and the sensing field. On behalf of the Prize Awarding Committee and the Editorial Board of *Sensors*, we would like to congratulate these eight teams for their excellent work. In recognition of their accomplishment, Drs. Ellen L. Holthoff, Xingwei Wang, Michele D. Kattke, Emilio Gil and Maria Luz Rodriguez-Mendez will receive 1,000 CHF, 800 CHF, 600 CHF, 400 CHF and 200 CHF, respectively, and the privilege of publishing an additional open access format paper of their choice, free of charge, in *Sensors* in 2015. Drs. Byoungho Lee, Alphus D. Wilson and Elizabeth A. Baldwin will be awarded the privilege of publishing an additional research paper free of charge in open access format in *Sensors*.

Prize Awarding Committee

*Editor-in-Chief*, Section ‘Physical Sensors’

**Dr. Vittorio M.N. Passaro**

Department of Electrical and Information Engineering, Politecnico di Bari, Via E. Orabona n. 4, 70125 Bari, Italy

Tel.: +39-080-5963-850; Fax: +39-080-5963-410

Website: http://dee.poliba.it/photonicsgroup

E-Mail: vittorio.passaro@poliba.it

*Editor-in-Chief*, Section ‘Chemical Sensors’

**Prof. Dr. W. Rudolf Seitz**

Analytical Chemistry, Department of Chemistry, University of New Hampshire, Durham, NH 03824-3598, USA

Tel.: +1-603-862-2408; Fax: +1-603-862-4278

Website: http://www.unh.edu/chemistry/faculty/seitz_w.html

E-Mail: wrs@cisunix.unh.edu

*Editor-in-Chief*, Section ‘Remote Sensors’

**Dr. Assefa M. Melesse**

Department of Earth and Environment, AHC-5-390, Florida International University, 11200 SW 8th Street, Miami, FL 33199, USA

Tel.: +1-305-348-6518; Fax: +1-305-348-3877

Website: http://faculty.fiu.edu/∼melessea/

E-Mail: melessea@fiu.edu

*Editor-in-Chief*, Section ‘Biosensors’

**Dr. Alexander Star**

Department of Chemistry, University of Pittsburgh, 219 Parkman Avenue, Pittsburgh, PA 15260, USA

Tel.: +1-412-624-6493; Fax: +1-412-624-4027

Website: http://www.pitt.edu/~astar/

E-Mail: astar@pitt.edu

*Editor-in-Chief*, Section ‘Sensor Networks’

**Prof. Dr. Leonhard M. Reindl**

Albert-Ludwigs-University of Freiburg, Faculty of Engineering, Department of Microsystems Engineering - IMTEK, Laboratory for Electrical Instrumentation, Georges-Koehler-Allee 106, Room 04-014, D-79110 Freiburg, Germany

Tel.: +49-761-203-7220; Fax: +49-761-203-7222

Website: http://www.imtek.de/professuren/emp/mitarbeiter/reindl/vita

E-Mail: reindl@imtek.de
